# Advancing the treatment of long-lasting borderline personality disorder: a feasibility and acceptability study of an expanded DBT-based skills intervention

**DOI:** 10.1186/s40479-022-00204-x

**Published:** 2022-12-12

**Authors:** Joaquim Soler, Elisabet Casellas-Pujol, Juan Carlos Pascual, Carlos Schmidt, Elisabet Domínguez-Clavé, Ausias Cebolla, David Alvear, Anna Muro, Matilde Elices

**Affiliations:** 1 Department of Psychiatry, Hospital de la Santa Creu i Sant Pau, Av. Sant Antoni Mª Claret 167, 08025 Barcelona, Spain; 2 Universitat Autònoma de Barcelona (UAB), Barcelona, Spain; 3 Institut d’Investigació Biomèdica-Sant Pau (IIB-NTPAU), Centro de Investigación Biomédica en Red de Salud Mental, CIBERSAM, Barcelona, Spain; 4 Departamento de Personalidad, Evaluación y Tratamientos Psicológicos, University of Valencia UV, Valencia, Spain; 5 CIBER Physiopathology of Obesity and Nutrition (CIBEROBN), Madrid, Spain; 6 Universidad del País Vasco/Euskal Herriko Unibertsitatea, leioa, Spain; 7 Department of Basic, Developmental and Educational Psychology, Universitat Autònoma de Barcelona Serra Húnter Programme, Generalitat de Catalunya, Barcelona, Spain; 8 Institute Mar of Medical Research, IMIM, Barcelona, Spain

**Keywords:** Borderline personality disorder, Long-lasting BPD, Feasibility, Acceptability, Skills training, Positive psychology, Dialectical behavior therapy, Contextual approach

## Abstract

**Background:**

Long-term follow-up studies in patients with borderline personality disorder (BPD) consistently show persistent impairment in psychosocial adjustment, although symptoms tend to decrease over time. Consequently, it might be better to deemphasize symptom-oriented interventions and instead promote interventions that incorporate patient perspectives on recovery. In this study we aimed to examine the feasibility and acceptability of a novel intervention (dialectical behavioral therapy combined with positive psychology and contextual-based skills) in the clinical treatment of long-lasting BPD difficulties.

**Methods:**

This was a qualitative study. We developed an initial 8-week group intervention for long-lasting BPD. Upon completion of the 8-week program, the participants were asked to participate in a group discussion to provide feedback. Based on that feedback, the intervention protocol was modified and then offered to a second group of patients, who also provided feedback. The protocol was revised again and administered to a third group. A total of 32 patients participated in the group interventions; of these, 20 provided feedback in the qualitative study. The main outcome measure was acceptability.

**Results:**

The following overarching themes emerged from the group interviews: helpful, unhelpful and neutral practices; internal/external barriers; facilitators; and effects. Participants reported difficulties in imagining an optimal future and self-compassion. By contrast, positive skills were associated with an increase in positive emotions. The main internal barrier was facing difficult emotions. The main external barriers were language-related issues. The group format was perceived as a facilitator to success. Dropout rates, which were assessed as an additional measure of acceptability, decreased substantially in each successive group, from 60 to 40% and finally 20%.

**Conclusions:**

The intervention was feasible to implement in the clinical setting and participants rated the final set of skills highly. Most of the skills were considered useful. Participant feedback was invaluable to improve the intervention, as evidenced by the large increase in the retention rate from 40 to 80%. Randomized clinical trials are needed to test the efficacy of this intervention in promoting well-being in participants with long-lasting BPD.

## Background

Borderline personality disorder (BPD) is a severe mental disorder characterized by pervasive emotion dysregulation, impulsivity, identity disturbances and conflicting interpersonal relationships [1, 8]. In the general population, the prevalence of BPD is around 2% [76], although it is substantially more common in clinical populations, with a reported mean point prevalence across studies of 28.5% [73]. BPD often presents with other mental disorders, including mood, substance use, and eating disorders, as well as post-traumatic stress disorder (PTSD) [1].

Despite the severity of BPD, remission rates within 10 years of diagnosis are as high as 85% [34, 77]. However, high remission rates do not necessarily indicate the absence of suffering or impairment, rather remission simply means that the patient no longer meets diagnostic criteria for BPD. Some authors argue that BPD progresses in stages, starting with an “at-risk” stage that eventually leads to clinical manifestations [42]. Clinical improvement appears to come at the cost of social isolation and a significant reduction in general activity. A relevant proportion of patients suffer from persistent impairment in psychosocial adjustment [2]. Follow-up studies have consistently found that some core BPD traits, such as impulsivity-related behaviors, resolve more quickly while other traits, such as those related to affective instability and anger, tend to be more enduring [54]. Crucially, only a small percentage of patients with BPD achieve remission of comorbid clinical conditions, most notable affective symptoms, which reflect loneliness, feelings of emptiness, and impaired psychosocial functioning [2]. In this regard, “long-lasting BPD” refers to patients that present some degree of clinical improvement, but in whom low mood, poor psychosocial adjustment, and feelings of emptiness persist over time.

In recent years, the concept of good mental health has been expanded to comprise more than simply a reduction in symptoms. Good mental health involves several domains, including meaningful living, the establishment of significant relationships and self-management strategies [27, 43, 46]. This broader conception of good mental health matches the perspective of patients with BPD with regards to recovery. Katsakou et al [45] conducted a qualitative study involving 48 patients with BPD receiving treatment in secondary mental health services. The interviews showed that patients associated recovery with an increase in several factors, including self-acceptance, self-confidence, control over emotions, better relationships, employment, and a decrease in symptoms such as suicidality and self-harm.

Psychotherapy is recommended as a first-line treatment for BPD [56, 72]. Several psychological interventions are available to treat BPD, although the most extensively studied and well-established approach is dialectical behavioral therapy (DBT), whose effectiveness has been demonstrated in multiple studies [8, 19, 72]. Standard DBT is a highly structured treatment based on cognitive-behavioral principles, integrating mindfulness and dialectical-based strategies into four different treatment modes (i.e., individual therapy, skills training [ST], phone contact, and consultations team) [49]. DBT offers a stage-specific treatment approach that is determined by the severity of the disorder. Treatment according to the stage, which ranges from stage 1 to 4, is important and necessary given the high complexity of BPD and related comorbidities. The main goal of stage one treatment is to reduce life-threatening behaviors (i.e., parasuicidal behavior, severe eating disorder and substance abuse). The focus of stage two is to help patients learn to experience emotions appropriately. Exposure-based strategies for treating intrusive symptoms, which may be related to comorbid PTSD, are recommended once the patient has improved stage one behaviors. Stage three treatments focus on improving problem-solving related to activities of daily living, increasing self-respect, and improving relationships. The aims of interventions for stage four include reducing the sense of incompleteness while increasing connectedness, and enhancing full participation in life, and acceptance [52].

Research in DBT has focused mainly on stage one (keeping patients alive) and stage two (emotional reprocessing) [8, 35, 52, 62]. Although follow-up studies suggest mood and interpersonal difficulties, evidence regarding stages three and four is scarce.

Studies on DBT skills training (DBT-ST) in BPD have evaluated the effectiveness of training four skill modules: mindfulness, emotion regulation, interpersonal effectiveness, and distress tolerance [50]. Those studies show that standard DBT (including all treatment modes) and DBT-ST mode alone are both effective in reducing self-injurious behavior, suicide attempts, and depression and anxiety [12, 48, 71].

Positive psychology (PP) has emerged as an interesting, evidence-based approach to improving well-being [12, 21, 47]. PP interventions focus on selecting and amplifying positive emotions, in some cases by replacing negative with positive emotions, based on the idea that positive experiences can attenuate or damper negative ones [65]. Within the PP framework, an individual who is capable of choosing how to act or what to pay attention to at a given point in time is more likely to act in a wiser, more virtuous manner, thus increasing well-being [67, 68]. Mindfulness is crucial to achieving those aims. Mindfulness and compassion-based interventions have been associated with increased positive affect, improved quality of life and pro-social behavior [4, 18, 20, 24, 28, 38, 59]. Some mindfulness practices, such as loving-kindness (LK) and self-compassion (SC), are part of a set of DBT skills named “other perspectives on mindfulness” [50]. LK and SC, along with mindfulness, have proven useful to alleviate suffering and promote well-being ([26, 33]; ​ ​[53, 57]). Nevertheless, most DBT skills training programs [10, 25, 50] focus on the “core mindfulness skills”, which teach participants how to practice mindfulness. Since LK and SC are not considered “core” skills, they are often left out of these programs as explicit teaching skills, even though some authors have suggested that they can be woven into the teaching of the “core” mindfulness skills [50]. Data from a pilot study showed that adding LK and SC practices to the traditional mindfulness skills resulted in greater levels of acceptance than mindfulness skills alone [26]. That study also observed significant improvements in BPD symptoms, self-criticism, and self-kindness [26]. A feasibility study performed to assess the value of PP exercises for patients with suicidal behavior found that exercises designed to increase gratitude and personal strengths were positively evaluated by the participants; conversely, forgiveness-focused exercises were rated poorly [39].

Although numerous studies have demonstrated the value of DBT for the treatment of stage one and two BPD, there is a notable knowledge gap regarding the value of DBT for stages three and four. Although some preliminary evidence suggests that positive psychology, loving-kindness and self-compassion exercises might be useful, more data are needed to support their use. In addition, published studies on patient perspectives on recovery in BPD are scant, and more data are needed to make treatment and outcome targets more relevant to patients.

In this context, there is a need to perform feasibility studies to determine whether an intervention is appropriate for further testing [9]. These type of studies are valuable because they reflect the reality of clinical practice, thus providing external validation by testing whether the feasibility of the intervention in real-world setting [9]. An important area of focus addressed by feasibility research is examining acceptability, which refers to participant perceptions of the novel intervention [9].

Accordingly, we performed the present qualitative study to examine the feasibility and acceptability of an expanded skills DBT-based intervention targeting long-lasting BPD. Acceptability was assessed through group discussions designed to obtain extensive feedback from the participants.

## Methods

### Participants

A total of 32 outpatients with long-lasting BPD participated in the novel treatment program (see below). All of these patients were recruited from the BPD unit at a public hospital in Spain. For the purpose of this study, “long-lasting” BPD was defined as the presence of some clinical improvement (no life-threatening behaviors or trauma-related symptoms for at least 12 months) accompanied by persistent low mood, poor psychosocial adjustment, and/or feelings of emptiness.

Inclusion criteria were: adults of either sex between 18 and 65 years of age; primary diagnosis of BPD according to DSM-IV-TR criteria and the structured Diagnostic Interview for Borderlines Revised (DIB-R) (Barrachina et al., 2004); previous participation in two DBT-ST interventions and signed informed consent. Exclusion criteria were: presence of life-threatening behaviors in the last 12 months; PTSD or related symptoms; diagnosis of drug-induced psychosis, organic brain syndrome, bipolar or psychotic disorder; intellectual disability; participation in any other psychotherapy treatment during the study. A subgroup of 20 participants participated in the qualitative study, which was conducted to assess the participants’ perception of the intervention.

### Procedure

First, we reviewed the available literature to identify interventions designed to treat long-lasting BPD symptoms. This review showed that PP exercises and compassion strategies seem to be useful for enhancing well-being [17, 26, 33, 47, ​^57^]. Consequently, we developed an intervention based on PP and contemplative practices (for more details see the Wellbeing Training Based on Contemplative Practices [WTCP]) manual: [13, 15, 26]). The skills trainers were directly trained in the WTCP protocol by the creator of that program. Next, we recorded a series of guided meditations aimed at facilitating the practice of the skills in the WTCP and we created a workbook describing the skills presented in the intervention. The intervention was led by three experienced therapists (all with PhDs) who had both research and personal experience in mindfulness practice. Two of the therapists (J.S and M.E) had received prior training in DBT, self-compassion programs, and CBT. The third therapist (A.M) had experience in CBT and PP.

We developed an initial 8-week intervention designed to treat long-lasting BPD. Ten patients were assigned to a group intervention. Upon completion of the 8-week program, the participants were invited to participate in group discussions to explore their experience with the training and the particular practices offered in those sessions. Two group discussion sessions were conducted by two independent researchers, who were not involved in the treatment of patients. The information obtained during these interviews regarding the value of each specific practice was then used to improve and refine the content of the intervention. The revised treatment protocol was then offered to a second group of patients, who also participated in a group discussion after completion of the 8-week intervention. This process was repeated to develop the third and final intervention, which included only the skills/practices considered most useful by most patients. Practices that had an aversive effect (i.e., increased distress) were discarded; only those practices considered useful by participants were retained.

The initial intervention was based on a combination of PP and mindfulness-based frameworks (see WTCP manual: [15]). The treatment format consisted of eight 2-hour sessions offered weekly in a group setting (minimum 10 participants). The structure of the sessions was similar to the DBT-ST format. At the first session, the norms and goals were presented and participants received the workbook and recorded meditations. The therapists emphasized that the goal of the intervention was not to achieve “instant” happiness, but rather to learn and practice evidence-based skills to promote well-being. Starting with the second session, a new skill was introduced each week. Practical exercises and/or a meditative practice were also practiced during the session. Participants were instructed to practice the skills learned at the session during the week. These skills and the homework assignments were reviewed at the start of the next session.

The first eight-week intervention was entirely based on the WTCP program [15]. This program included training in the following skills: 1) Best Possible Self (participants were instructed to write a letter about the best version of their self-projected self over the next 10 years); 2) Mindfulness (breath and body meditations); 3) Identification and development of personal strengths; 4) Empowering positive emotions (savoring and gratitude); 5) Regulating negative emotions; 6) Contribution (compassion and altruism); and 7) Multi-Self (including compassionate self). In this first intervention, most participants struggled with the concepts of “happiness” and “self-compassion”. In addition, most of the patients considered the practice of projecting oneself into the long-term future (i.e., Best Possible Self strategy) to be aversive. Only four of the ten participants completed the entire 8-week intervention, a 60% dropout rate.

To address this high dropout rate and dissatisfaction with certain elements of the intervention, the intervention was modified based on feedback from the participants. The “My best possible self” skill was reduced to 1 year (instead of 10) and we also modified the sequence of skill presentation. In addition, during the post-intervention interviews, participants reported experiencing high levels of distress during the intervention. Consequently, we moved the presentation of self-compassion skills to session 1, and also extended training in self-compassion to four sessions. The “regulating negative emotions” model session [15] was replaced with self-compassion strategies. Instead, participants were encouraged to practice the skills covered in the emotion regulation module of DBT-ST. References to “happiness” were avoided.

A second group of patients (*n* = 10) that met the inclusion criteria were recruited from the BPD Unit to participate in this revised intervention. Upon completion of this second 8-week intervention, feedback from the participants confirmed difficulties with the “Best Possible Self” practice. Consequently, we decided to eliminate this skill in the next version of the protocol. Participants also reported difficulties with the concept of “self-compassion”, which some perceived as sorrow or self-pity. Importantly, six of the ten participants completed full intervention, a dropout rate of 40% (versus 60% in the first group). Both patients and therapists agreed that eight sessions were insufficient to introduce and train all of the content.

Based on these data, the protocol was again revised and expanded from eight to 12 weekly sessions. The revised protocol included new strategies to work on self-compassion as a form of self-care. In terms of sequence of skills, the revised protocol began with self-care skills, based on the observation that a minimum degree of loving kindness and compassion towards the self is essential before training in positive emotion strategies. These self-care skills were based on the DBT practice of “Please Self-care” (see PLEASE skills: [49, 50]), which consists of practicing self-compassion by performing “self-care acts”, defined mainly as behaviors involving taking care of the body under the premise of “*fake it until you make it”.*

In addition, we detected a need for strategies to defuse cognitive barriers for self-care training. Consequently, we developed and included a new module, which we called “Cognitive Self-Care”, to address different polarities of thought. Defusion strategies played a key role in these practices. Additionally, we incorporated a practice called “Wise-Matrix”, which combines “the Matrix” from Acceptance and Commitment Therapy (ACT) [60] and the “Wise Mind” skill from DBT [50]. The aim of this combined practice is to promote new perspectives for problematic thoughts and emotions to lower emotional reactivity. Most patients rated the savoring and gratitude practices as useful and thus these were retained. This third and final intervention group had the lowest drop-out rate of the three groups (20% vs. 40 and 60%). Ten patients out of twelve completed the full intervention. All the practices and skills used in the final intervention are described in Table 1.

**Table 1:** Summary of the final 12-week intervention: skills and strategies and order of presentation

Session number and content/goals	Skill or Strategy	Description
1. Introduction	Emphasis on self-care: biology, behavior, thoughts, and emotions	Presentation of the goals of the intervention as well as the norms, framework and skills trained.
2. Activating and maintaining self-care behaviors	Please Self-care	The aim of this skill, from DBT (see PLEASE skills: [49] [50];), is to facilitate behavioral changes through self-care. It consists of five components: (i) addressing physical illness; (ii) balancing eating; (iii) avoiding mood-altering substances such as alcohol, drugs, non-prescribed medications or misuse of prescribed medications; (iv) balancing sleep; and (v) exercising.
3. a) Identify internal and external barriers that keep us away from what is important to us	Wise-Matrix	The Matrix is a therapeutic tool created by Polk and Schoendorff [60] and used in the context of ACT [36] to provide a contextual view of problematic behaviors. The aim is to explore the following: values, obstacles/barriers, inflexible behaviors, and valued actions. This tool is useful to generate cognitive defusion, although it can generate elevated distress when applied in patients with severe BPD, especially when internal obstacles are identified. To avoid distress, participants were instructed to answer the MATRIX questions from the DBT “wise mind” skill ([49], [50]), which helps to generate a defused perspective of inner experience while avoiding the extremes of “emotional mind” and “rational mind”. This skill was named “Wise-Matrix”, as it combines both strategies.
b) Orient towards values and define goals to be achieved	Values	Based on the emotion regulation skills of DBT (see Emotion Regulation Handouts: [50], p. 388), participants were asked to answer the question “*what is most important to me right now?”*, to identify their main values. Once these values were identified, goals and concrete actions were defined to help the participants put each value into practice on a daily basis.
4. Identify personal strengths	Personal Strengths	The freely available VIA *Character Strengths Survey* (https://www.viacharacter.org) was used to identify personal strengths. This questionnaire assesses 24-character strengths classified into six broad categories of virtues: wisdom, courage, humanity, justice, temperance, and transcendence [61]. Participants were told to complete the questionnaire and to identify their five main strengths. The main goal of this strategy was to define and generate behaviors linked to personal strengths. For example: *“activating the strength of kindness by acting kindly towards co-workers”.* As a way of motivating concrete actions, the personal strengths were associated with the values previously identified in session 3.
5. Identify levels of self-care: cognitive	Cognitive Self-care^a^ Loving kindness^a^	The goal of this session was to foster the understanding of the importance of self-care through loving kindness. This skill focuses on identifying polarities of thought (i.e., *“fusion* vs. *defusion”, “kindness* vs. *hostility”, “shared humanity* vs. *isolation”, “impermanence* vs. *permanence”* ...) in order to generate more flexible ways of relating to one’s own experience. Some of the main targets are to generate perspective, identify forms of experiential avoidance, facilitate a connection with others, and to foster acceptance that the world, like our experiences, is changeable. The goal is to defuse rigid rules such as “*my life will always be the same*” or “*I can’t trust others”.*
6. Identify levels of self-care: behavioral	Body Self-care	The main target of this session is for participants to learn to calm themselves as a prelude to compassion. This skill consists of using body-related practices to facilitate self-compassion and self-calming. The skills used were “half smile” and “willing hands” (DBT: [49] [50];), “compassionate gesture and tone”, and “calming touch”.
7. Identify levels of self-care: emotional	Self-compassion	The aim of this session is to appreciate the difference between self-care and self-criticism, as well as how to use meditations to facilitate self-compassion (e.g., *“safe place imagery, compassionate color, interconnection meditation …”*). Some of the meditations are available in many mindfulness-based interventions (e.g., MSC: [30]). The goal is to facilitate self-care skills and a kinder relationship with one’s own experience. Participants were encouraged to practice self-compassion by talking to themselves as they would talk to a loved one. They were also encouraged to identify self-critical phrases and to replace them with self-compassionate ones.
8. a) Identify internal parts of the mind (multi-self). b) Generate a perspective of the different multi-self.	Multi-Self	This strategy is widely used in compassion-focused therapy (WTCP; CFT: [31]: [15]). It consists of exploring the possible multiple selves that can appear in the same situation and their relationship with different emotions such as anger, anxiety, and sadness. The goal is to show the participants that there are different possibilities of experiencing a situation and not just a “single self”. This skill allows the participants to practice perspective-taking to create “a compassionate self”.
9. Enhancing positive emotions	Savoring	To practice intensifying and prolonging positive emotions in a deliberate way. Participants can be trained in savoring by increasing awareness of something positive from the present, remembering something from the past or anticipating something good in the future [5, 6, 64]. Listing their favorite experiences using the five senses and then experiencing them in daily life or bringing this to mind during a meditation practice were examples of savoring-training. Specific tasks were: 1) Identify favorite sensory experiences; 2) Identify thoughts and behaviors that boycott positive moments; and 3) Record three positive events in the day and savor them.
10. Enhancing positive emotions I	Gratitude	The goal of this skill was to pay attention to valued things in daily life that are not usually appreciated. For example: *“Find three things during your day that you are grateful for and savor them for 20-30 seconds”*. These practices included: thinking about supportive relationships, what others have done for us, a gift you have appreciated, feeling grateful for something or to someone … Participants were also asked to make a record of self-critical or judgmental thoughts and then turn them into a gratitude sentence.
11. Enhancing positive emotions II	Contribution	This practice consists of taking concrete actions to help others (i.e., “*perform 5 kind acts during the week*”). The aim is to increase self-esteem through helping loved ones and nurturing old and new relationships. This skill is commonly used as a distraction and distress tolerance strategy in DBT ([49] [50];). Contribution allows individuals to redirect attention from the self to others and can be useful to interact with others.
12. Motivate daily practice of learned skills	Summary of skills and strategies learned	The final session focuses on underscoring the importance of continuing to practice the skills learned. Suggestions are given on how to facilitate practice in daily life.

^a^*Note*: The terms self-care and loving kindness were used instead of “self-compassion”

### Data analyses

In this study, we used a mixed-methods approach, which consisted of a quantitative description of the participants’ characteristics and attendance rates, together with a qualitative description of the participants’ experience with the intervention obtained through group discussions led by experienced clinicians.

We evaluated the opinions and experiences of the participants as a result of the process. We obtained qualitative data by means of a clinician-led discussion within the established groups. These discussions were based on a series of semi-structured questions designed to investigate the participants’ experiences around six different themes established by the research team (these themes were selected prior to conducting the group discussions) (Table 2). First, participants were asked to give their opinion on each theme. If any contradictory statements or disagreements emerged, the discussion moderator asked the participants to clarify their statements. The data obtained in the discussion groups were analyzed using an interpretative phenomenological approach (IPA [];). Since the individual’s perspective cannot be directly accessed in IPA, the researcher interprets the participant’s interpretation through a double hermeneutic process [23]. This process allows the researcher to reflect on the participant’s insider perspective in the broader context, informed by scientific publications and research on the topic/experience under study. Following IPA guidelines [70], the participants were encouraged to express themselves freely; when necessary, the researcher asked for clarification. The researchers encouraged participants to express their opinions as naturally as possible. The recorded discussions were transcribed verbatim and were read several times, line-numbered, and organized by codes into subthemes [23]. Disagreements between the two researchers were resolved by consensus between the researchers and the first author. Analysis was supported by Atlas.ti, (v.22).

**Table 2:** Topics and example questions

Topic	Definition	Example questions
Helpful practices	Practices associated with increasing positive emotions and/or self-awareness; and/or decreasing negative emotions and self-criticism.	*Of all the practices and exercises we have done during the course, which were helpful (if any)?*
Unhelpful practices	Practices identified as unhelpful because they increased negative emotions, and/or generated self-criticism.	*Of all the practices and exercises we have done during the course, which were unhelpful (if any)?*
Neutral practices	Practices not related to any particular effect.	*Of all the practices and exercises we have done during the course, which had a neutral effect (*i.e.*, neither helpful or unhelpful)?*
Barriers	External and/or internal factors that interfered with the learning process and practice.	*In your opinion, did anything interfere with your learning process* *(*e.g.*, the group format, the therapist’s embodiment of the skill, your previous training …*)?
Facilitators	External and/or internal factors that facilitated the learning process, skills-training, and the homework assignments.	*In your opinion, did anything facilitate your learning process* *(*i.e.*, the group format, the therapist’s embodiment of the skill, your previous training …)?*
Effects	Consequences related to the intervention. Internal effects on mood, self-awareness and/or self-dialogue. External effects on social functioning.	*Did you experience any changes during the training?* *Has the course had any effect on you?* *Has anything changed for you since you finished the course?*

## Results

### Sample characteristics

A total of 32 outpatients participated in the group interventions. Most patients (95%) were female. The mean age (standard deviation [SD]) age was 40.3 (6.1) years (range, 27-45). Twenty of these patients were interviewed in the post-intervention feedback groups. The educational level was as follows: university studies (56.3%), secondary studies (37.5%), and primary education (6.3%). At the evaluation, 37.5% of the sample was unemployed, 31.3% were receiving an invalidity allowance, and 25% were employed. Most patients (75%) were single. Most patients lived alone (43.8%) or with their birth family (31.3%).

### Participants’ experiences

Six overarching themes were explored in each group discussion, as follows: 1) helpful practices, 2) unhelpful practices, 3) neutral practices, 4) barriers, 5) facilitators, and 6) effects. Later, several sub-themes were identified based on an analysis of the transcribed text of the discussions (Table 2).

A central theme was the distinction between helpful, neutral, and unhelpful practices, which was based on the participants’ experience with these different skills during training. Among the helpful practices, practicing gratitude and savoring, and mindfulness were associated with an increase in positive emotions in 15 of the 20 participants (75%), as evidenced by the following comments:

*“I noticed that being grateful for the things that I have lifts my mood. At night, instead of thinking about everything that goes wrong, I do the gratitude practice, and I feel much better for all the good things I do have in my life”.*

*“It has helped me to accept that I will have negative feelings, and that I can feel the pain and continue with my life (mindfulness with emotion)”.*

Some participants (6/20; 30%) reported that completing the Strengths Questionnaire was helpful to become more aware of their strengths. The questionnaire helped raise awareness of some personal characteristics that they had not noticed before or shifted how they perceived some of these characteristics.

*“I already knew I had these characteristics, but perhaps I did not see them as strengths. There are things that people say about me that I do not believe. The questionnaire has helped me to see them as good things about myself”.*

For some, being more aware of their strengths was also related to decreasing self-criticism:

*“According to the questionnaire, some of my strengths are equanimity, honesty, and appreciation of beauty and excellence. At first, I could not believe it because I have always been in touch with the negative side. Now, I do not see myself as bad as I used to”.*

In addition to identifying these personal strengths, a substantial proportion of participants (12/20; 60%) noted that practicing self-compassion and loving-kindness had a positive impact:

*“I am so harsh on myself. Compassion practices help me notice that and decrease the number of self-judgments, which has an impact on the way I see others. I do not judge them as much either”.*

*“For me, the best thing has been the work with self-criticism, it has helped me a lot to use the third person, to ask myself if I would speak to another person like that”.*

*“Combining self-compassion talk with touch helps me. It didn’t help me as much to tell myself things as it would to someone else, but to put a hand on my chest and feel myself, helps. If I just tell myself I don’t believe it, then the reassuring touch helps me believe it”.*

Finally, most participants (7/10; 70%) rated the “Wise-Matrix” as a useful but complex tool:

“*Just realizing what is the internal world and what is the external world is what helped me the most. Understanding it, integrating it and practicing it is the most important thing. I wouldn’t have been able to stand being in this group if it hadn’t been for the matrix, because my heart is going super-fast and I can still stay here”.*

*“The matrix has placed me in the world, it has made me more aware of what I had in my head and believed to be real. Now I can distinguish the difference between my inner world and the outside world”.*

*“The matrix is very useful, but I have to keep working on it. When something affects me, I wonder if it is something from the outside world or from the inside world. Now I see that difference, but I still find it hard not to believe what my mind is telling me. I also am more aware of whether or not I am moving away from what matters to me. For example, with interpersonal relationships, if I am very vulnerable, I prefer to take a step back and face another day, instead of saying or doing something that is going to be bad for the relationship”.*

By contrast, some of the practices were considered unhelpful. For example, being more aware of particular internal processes was associated with an increase in negative affect.

*“Trying to practice self-compassion was awful for me because I became aware of how much I criticize myself, not three times a day, but 20 hours a day. This made me feel like some monster, and it was unpleasant for me; it made me angry. It is tough for me to practice self-compassion".*

In addition, the practice of writing a letter to the future self was perceived as bitter-sweet. While some participants (3/10; 30%) considered it to be a sort of “roadmap” for the future, for most (7/10; 70%) the experience of projecting themselves into the future was unpleasant and triggered anxiety.

*“I do not see the point in writing the letter. I find it difficult to survive each day, so I cannot make a plan for the next six months”.*

In relation to internal barriers, many participants (9/20; 45%) identified “moodiness” as the primary obstacle to practicing the skills presented:

*“Some days, I am just not open to this. On those days, having to find things to be grateful for is negative. If I have a bad day, I prefer to deal with it rather than feeling forced to find good things in my life”.*

*“When I feel “normal”, practicing is not a problem. However, when I am having a bad day, it is impossible for me to apply self-compassion. I wish I could, but I simply cannot”.*

In terms of external barriers, some participants (4/20; 20%) identified the characteristics of the group leader as not being helpful. A few participants (3/20; 15%) also criticized how the content was presented, suggesting that it would be better to use a more structured presentation during the session, with a booklet to review the content at home. Most participants underscored difficulties in the use of concepts such as “happiness” and “self-compassion” during the training.

*“Sometimes I thought that the group leader was oversimplifying this and was being condescending. We have particular problems as a group. We deal with pervasive emotion dysregulation, and the leader must acknowledge that”.*

With regard to facilitators, most participants (12/20; 60%) pointed to the value of the group format, as seeing others experiencing difficult times helped to validate their own feelings. Some participants also noted that the therapist’s ability to “embody” these practices and to direct the group to prevent deflection from the session’s subject.

*“You can tell that the therapist practices these skills … and I wanted to be a little bit like him, if only just a little!”*

The role of practice was also considered pivotal to integrating the concepts learned during the sessions. A high proportion (15/20; 75%) of participants highlighted the importance of having previously attended DBT-ST before being introduced to these new skills:

*“Five years ago, I would not have had the necessary skills to understand these. I could neither understand nor regulate my emotions. I was in a very dark place”.*

*“Some years ago, I felt that I was living in a storm most of the time. If somebody had said to me `look at the sun`, I would not have been able to do so. I would have thought: what are you talking about? Some years ago, the content of these practices would have been impossible for me to understand”.*

Lastly, in relation to the effects of the intervention, several participants perceived a decrease in self-judgment upon completion of training:

*“Now that we have finished the training, I notice that I am more kind to myself … I have to work on this a lot, but I am starting to see the effects”.*

An increased capacity to notice small things that happened during the day was also relevant for many:

*“I have learned that good things happen to me despite feeling pain, so I can feel bad about something and still appreciate good things in my life …”*

The effects of the practices were also evident in terms of interpersonal relations, with participants finding themselves to be “less reactive,” “less judgmental,” and “more compassionate” towards others:

*“Being more self-compassionate and more in touch with positive emotions is something that has not only helped me, but it has kind of expanded to other people in my life”.*

*“My daughter and I do not get along. She is going through adolescence, and now I am more aware of the details. I sometimes notice that she does things that make me happy”.*

**Figure 1:**
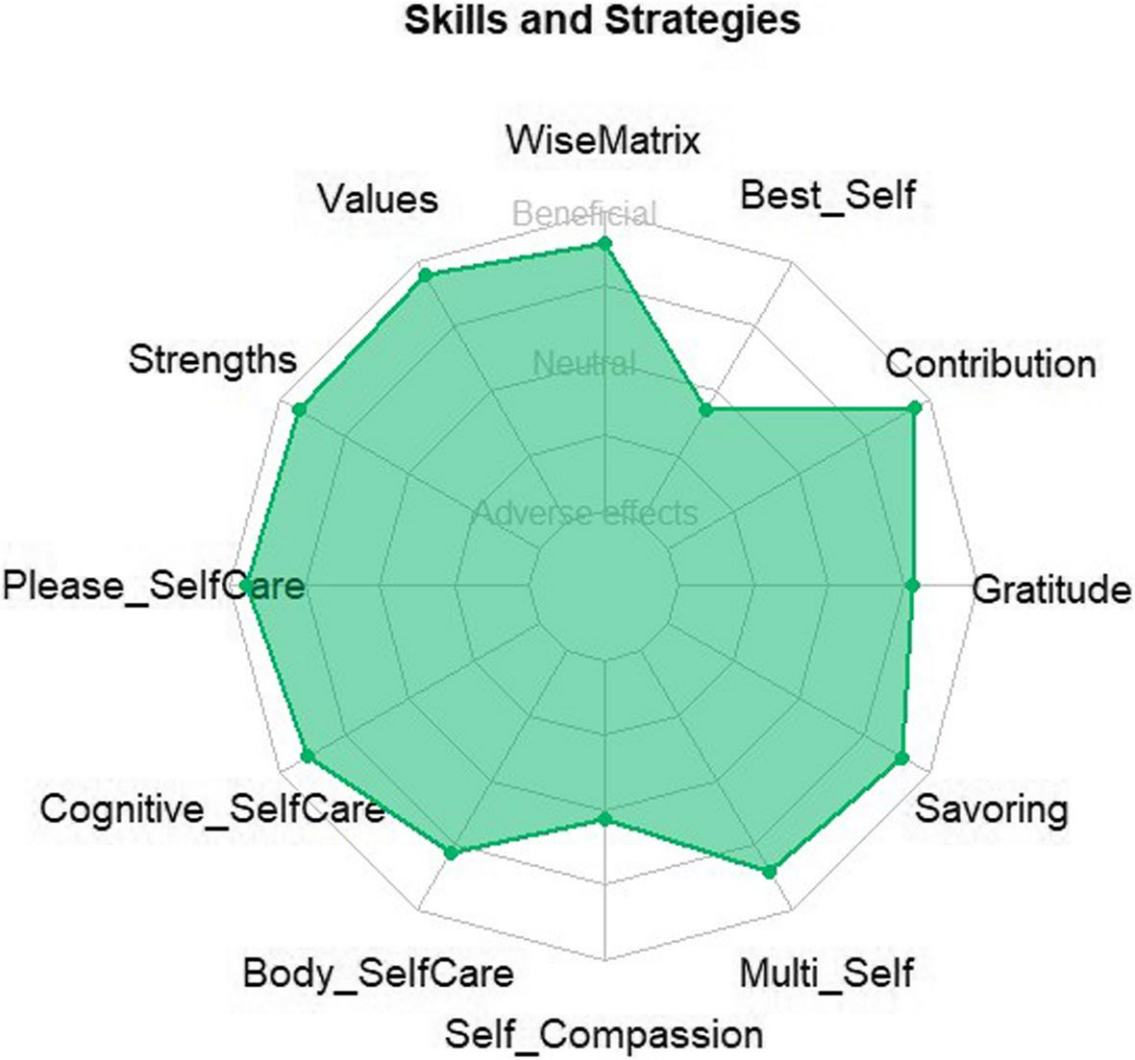
Fig. 1

Based on the participants’ feedback, the researchers rated each skill and strategy used in the group interventions as useful (or beneficial effects reported), neutral, or not useful (or adverse effects reported). Fig. 1 shows the mean rating of each skill and strategy in terms of their perceived effects. On average, the skills related to self-compassion and the My Best Possible Self strategy were evaluated as neutral, while the other skills were better accepted and generally rated as useful.

## Discussion

This qualitative study was performed to examine the feasibility and acceptability of an add-on skills training intervention involving the combination of DBT-ST, positive psychology, and contextual-based skills. This combined intervention was implemented in a clinical setting to treat long-lasting BPD in a group of patients who had previously undergone DBT-ST. The intervention was feasible to implement in this real-world setting and participants rated the final set of skills highly. Most of the practices were considered useful.

DBT-ST primarily addresses the first stage of DBT treatment, mostly focusing on reducing life-threatening behaviors and provide the emotional regulation and tolerance required in order to then shift the emphasis to trauma-related symptoms in stage 2. In this regard, strategies that facilitate the transition to stages 3 and 4 of DBT in patients with long-lasting BPD are needed, with a focus on enhancing connectedness and reducing the sense of emptiness. Positive interventions could help to promote both of these aims. A strategy designed to increase positive emotions would seem appropriate once behavioral decontrol has been overcome when further clinical improvement may come at the cost of social isolation, low mood, and generalized avoidance [2]. Accordingly, we developed and implemented a pilot PP-based intervention (WTCP [15];). Given the need to align therapeutic goals with patient perspectives on personal recovery, we used interview groups to obtain feedback from participants, and then used this information to progressively adapt and improve the intervention by eliminating the least useful practices while retaining the practices that participants considered helpful.

### Positive skills

For most participants, practicing positive skills such as gratitude, savoring, and working with personal strengths was associated with an increase in positive emotions, in line with the findings described by [39]). This improvement in positive emotions is consistent with other studies that have shown an association between savoring and decreases in negative affect and self-reported depressive symptoms [40], and between savoring and increased happiness and satisfaction [41, 44]. Previous studies have also found that gratitude has positive effects in non-clinical populations by reducing repetitive negative thinking [37], increasing positive emotions [22], and potentially even direct reducing suicidal ideation [48]. Interestingly, practicing these positive skills not only had a beneficial effect on the participants’ emotions and self-awareness, but they also had a positive influence on interpersonal relationships. Overall, the participants reported a decrease in self-judgment and an increase in their ability to notice small positive things. Participants also reported being less reactive and more compassionate towards others. Overall, the effects of these practices are aligned with important areas of recovery as suggested by [45]). Hence, it appears that positive strategies (e.g., savoring, gratitude, and personal strengths) may enhance the expected effects of compassion-related skills to target the specific problems underlying long-lasting BPD.

Despite the potential benefits of positive psychology for both nonclinical and some clinical populations [11, 16], the application of some of these strategies in patients with BPD may not be suitable. For example, we found that imagining an optimal future was unhelpful, in line with the findings reported by Huffman et al [39] in a sample of suicidal patients. Similarly, projecting a future self was problematic for some of the participants in our sample.

### Self-compassion skills

Self-compassion exercises also triggered difficult experiences, which some patients found hard to regulate when they became aware of their self-criticism. Interestingly, this finding contradicts preliminary evidence from other studies that suggest that compassion exerts helpful effects in patients with personality disorders [26, 51]. However, the difference in these results could be attributed to the study cohort, which involved a mixed sample of patients with personality disorder. Although training in self-compassion fosters positive emotions in the long-term, unpleasant emotions (especially grief, shame, and anger) are likely to emerge during the training period, a phenomenon known as “backdraft” [29, 75]. This “unwanted effect” is similar to those reported in mindfulness-based interventions [14], which seem to be transitory.

### Sequence of skill presentation

Although the sequence of skill presentation in DBT has not been extensively studied, future studies are needed to investigate this aspect in more detail. Elices and colleagues [25] found that patients with BPD who participated in DBT training were significantly more likely to drop out of treatment when the first skill taught was mindfulness versus interpersonal effectiveness (IE). Those authors hypothesized that participants may have perceived mindfulness skills to be more anxiety-provoking and less connected to their treatment goals than IE, which would explain the higher and faster dropout rate. Interestingly, the patients who received mindfulness training first and did not drop out showed greater improvement in BPD symptoms compared to those in whom the sequence of training started with IE skills; in addition, relevant changes, such as reduction of judgmental stance and defusion of self-critical thoughts, were only achieved throughout the mindfulness condition [10, 25, 63]. Based on our experience, we believe that self-compassion can cause short-term discomfort (similar to that observed with mindfulness), but this practice is likely to reduce self-invalidation, a core feature of BPD, in the long-term [26].

Self-criticism and self-invalidation [49] refer to two different but overlapping concepts. Given that self-invalidation is a core aspect of BPD and that both self-criticism and self-invalidation are cause and consequence of high emotional vulnerability that handicap patients’ capacity for joy, it would seem appropriate to target these areas. A recent review found that compassion-based interventions reduce self-criticism and that longer interventions were associated with greater effect sizes [74]. Consequently, it is crucial to find ways to ensure that patients continue with the intervention for a sufficiently long period to notice the benefits, despite the transitory backdraft. In this context, the sequence in which different skills and practices are presented appears to play a key role. For example, training loving-kindness before compassion appears to be beneficial, especially in participants with long-lasting BPD because loving-kindness training increases positive emotions and fosters greater openness towards other practices, such as compassion. Our experience suggests that the transition between the two practices is easier than if offered in reverse order, but more research is needed to test this hypothesis.

### Barriers and facilitators

In our study, some participants described “moodiness” as an internal barrier to practice. As some participants noted, they did not always feel “open” to the practice, which suggests that a certain degree of emotion regulation may be necessary before practicing certain skills. In this context, it is worth noting that all of the participants had previously received DBT-ST before enrolling in the current study. As a result, their prior training provided them with the personal resources necessary to face distressing emotions provoked by new skills. Several internal barriers to practice were detected, making these potential targets for future studies. Indeed, defusion strategies were needed to address these barriers, mainly the “wise matrix” tool which we developed for this intervention.

External barriers were mostly related to the attitude of group leaders and language-related issues. For example, the use of concepts such as “happiness” and “self-compassion” had a negative impact. Talking about “increasing happiness” evoked unrealistic expectations, which was experienced by some participants as something that “cannot be achieved”. As a result, this caused frustration and was perceived as invalidating for most of the participants. The main “facilitating” factor was the group therapy format, as it can reduce feelings of being “weird” or “alone”.

### Strengths and limitations

This study has several limitations. First, the sample was predominantly female and Caucasian, and all participants had prior experience with DBT-ST. In addition, only the participants who had completed the full intervention took part in the post-treatment group discussions. Unfortunately, as occurs in many qualitative research studies, we were unable to explore the experiences of the participants who dropped out. In addition, due to the preliminary study design (aimed at assessing feasibility and acceptability), we did not use validated measures to assess the impact of the intervention on different domains. Similarly, we did not track home practice, and therefore, we cannot make any definitive claims about the association between adherence to practice and the effects thereof. We used a series of broad questions to elicit a wide range of responses. As a result, the participants’ statements about the intervention were spontaneously reported. We purposely did not ask about specific skills, as we assumed that skills not mentioned were neither helpful nor unhelpful. In retrospect, it might have been better to specifically ask about each skill and it would probably be beneficial in future studies to do so. Despite these limitations, the study also has several important strengths. First, the findings broaden our understanding of the feasibility and acceptability of a positive-contextual psychology skills training intervention in patients with long-lasting BPD. This study also represents an important first-step towards facilitating the transition to stages 3 and 4 of DBT, and towards developing a targeted program whose efficacy could be evaluated in future research studies. In addition, by examining the specific skills rather than the final “package”, we increased our understanding of the processes of change in psychotherapy, which is congruent with new approaches in the field such as the process-based framework [16].

## Conclusions

In this qualitative study, we examined the feasibility and acceptability of a novel 8-week intervention designed to treat long-lasting BPD. The intervention was feasible to implement in the clinical setting and the final set of skills was highly rated by participants, who considered most of these strategies/skills to be useful. By using the participants’ feedback to modify the intervention, we were able to substantially improve retention rates (an additional measure of acceptability), which doubled from 40% in the first group to 80% in the final group. More research is needed to test the efficacy of this intervention to promote life fulfillment in participants with long-lasting BPD.

## Data Availability

The dataset and materials analyzed during the current study are not publicly available.
